# Molecular Epidemiology of HIV-1 among Prisoners in Central Brazil and Evidence of Transmission Clusters

**DOI:** 10.3390/v14081660

**Published:** 2022-07-28

**Authors:** Tayana Serpa Ortiz Tanaka, Gabriela Alves Cesar, Grazielli Rocha de Rezende, Marco Antonio Moreira Puga, Sabrina Moreira dos Santos Weis-Torres, Larissa Melo Bandeira, Maurício Antônio Pompílio, Julio Croda, Monick Lindenmeyer Guimarães, Ana Rita Coimbra Motta-Castro

**Affiliations:** 1 Laboratory of Clinical Immunology, Federal University of Mato Grosso do Sul, Campo Grande 79070-900, Brazil; g.alves.cesar@gmail.com (G.A.C.); grazielli_r@hotmail.com (G.R.d.R.); marco.m.puga@gmail.com (M.A.M.P.); weistorres.sms@gmail.com (S.M.d.S.W.-T.); mapomplio@yahoo.com.br (M.A.P.); juliocroda@gmail.com (J.C.); 2 Fiocruz Mato Grosso do Sul, Fundação Oswaldo Cruz, Campo Grande 79081-746, Brazil; 3 Laboratório de AIDS e Imunologia Molecular, Instituto Oswaldo Cruz, Fundação Oswaldo Cruz, Rio de Janeiro 21040-360, Brazil; monicklg@ioc.fiocruz.br

**Keywords:** HIV, vulnerable population, molecular epidemiology, drug resistance mutations, transmission network

## Abstract

Higher rates of human immunodeficiency virus (HIV) infection have been detected in prisoners when compared with the general population, but research into HIV molecular epidemiology and its transmission network has been lacking among them. Thus, this study aimed to verify potential HIV molecular transmission networks among prisoners. In addition, we aimed to describe the mutations related to antiretroviral resistance in these isolates. Thus, we conducted a cross-sectional survey from 2013 to 2018 in prisons in Central-Western Brazil, and the final sampling composed of 84 prisoners. Proviral DNA was extracted from each whole blood sample followed by amplification of the partial *polymerase* gene and sequencing. Forty-nine sequences (58.3%) were classified as subtype B, followed by C (14.3%), D, and F1 (2.4% each). A complex and dynamic HIV-1 epidemic is observed in the prisons, as 25% of the sequences were recombinant forms. We detected 15 HIV transmission clusters composed of at least two sequences, that included not only prisoners but also individuals from the general population from the same State with a variety of risk behaviors. Thirty-two percent (32.0%) of treatment-experienced prisoners had at least one drug resistance mutation (DRM), while transmitted DRMs were found in 5.9% of the prisoners. We highlight the urgent need for routine surveillance of HIV-1 infection including resistance genotypic tests considering the high disease burden, risky behaviors inside prisons, and the dynamic relationship of prisoners with the outside community.

## 1. Introduction

The prison population has increased by 175% in South America between the years 2000 and 2020 [1]. Brazil has the third-largest prison population in the world, with 811,707 individuals incarcerated in penal institutions, with a high prison population rate, increasing annually over the last 18 years [2]. The state of Mato Grosso do Sul (MS), Central Brazil, has the highest rate of incarceration mainly due to drug-trafficking crimes. In November 2021, the total prison population in MS State was 20,189 inmates, while the capacity of MS State’s prison system was only 10,910 inmates [3], thus demonstrating the overcrowding that could have a significant impact on sanitary and health conditions.

In Brazil, screening for human immunodeficiency virus (HIV) infection may be recommended for prisoners who present high-risk behaviors. Counselling, treatment, and care should be part of an HIV program aimed at improving health care in prison and at making it correspond to that offered to the public [4]. Currently, though, these objectives are largely unmet because of the Brazilian penitentiary system’s limitations, such as severe budget restraints leading to under-resourced prison settings, prison overcrowding, deprived condition of the existing health facilities, underpaid prison staffing, recurrent inmate transfer between prisons, and particularly the low offering and execution of screening tests for HIV [5,6].

Once diagnosed, HIV infection should be treated using antiretrovirals (ARV), which are freely available to all HIV-infected persons in Brazil, as well as to prisoners, in the public health system. However, the prisoners usually present low adherence to treatment leading to virologic failure, as demonstrated previously [5,7], which reveals the inadequacy of the public health programs, especially as they apply to key populations (KP) such as inmates. In Brazil, a genotypic resistance test is recommended only if the following criteria are met: regular use of ARVs for at least six months; confirmed virologic failure in two consecutive HIV viral load (HIV-CV) tests, with a four-week interval between them; HIV-CV greater than 500 copies/mL [8].

HIV infection is almost ten times more prevalent in prison than in community settings [4]. As there is no countrywide prison surveillance system in Brazilian detention centers, data on HIV prevalence, the prevalence of HIV resistance mutations, and HIV transmission networks in prisons are limited to observational surveys. There is considerable variation in the estimates reported by them, most were derived from studies in only one prison, and HIV-1 prevalence ranged from 1% to 16% [6,7,9,10,11,12,13,14,15,16,17,18,19].

As the prevalence of HIV infection is higher in prisons, this high rate may reverberate throughout the community because prisoners have regular sexual intercourse with their partners living outside the prison (a legal right of Brazilian prisoners) [20]. In addition, the risk of transmission is favored by the high rate of prisoners’ movement between penal institutions and the high rate of reoffending, leading to return to prison, and thus establishing HIV transmission networks not only within prisons but between prisoners and the general population.

One way to investigate the existence of HIV transmission networks is to use phylogenetic analysis. Phylogenetic and phylodynamic approaches are broadly used to update HIV epidemiology, infer changing trends in the local and regional spread of HIV-1, and guide prevention measures and public health interventions [21,22,23]. This study aimed to verify potential HIV transmission networks among prisoners by investigating HIV polymerase nucleotide sequences isolated from HIV-infected prisoners and those obtained from other populations in MS, available in sequence databases. In addition, we also aimed to describe the mutations related to antiretroviral resistance in these isolates.

## 2. Materials and Methods

### 2.1. Subjects and Study Design

We conducted a cross-sectional survey, using information from databases, samples from biorepository, and active searching, with the completion of laboratory analyses. This study was conducted among prison inmates from 2013 to 2018. Whole blood samples were collected in two previous cross-sectional studies [6,24] and also from an active search. In the first study, twelve prisons were selected from the five largest cities in the MS State. Proportional stratified sampling was performed, and 1.6% (54/3362) of the prisoners had detectable anti-HIV antibodies in serum samples [6]. In the second one, involving two prisons, the HIV prevalence was 6.8% (19/279) [24]. The active search consisted of periodic visits, from May to July 2018, at various prisons in the capital of MS state, with the primary objective of identifying new HIV cases among prisoners who did not have blood samples collected in the previous studies.

**Figure 1:**
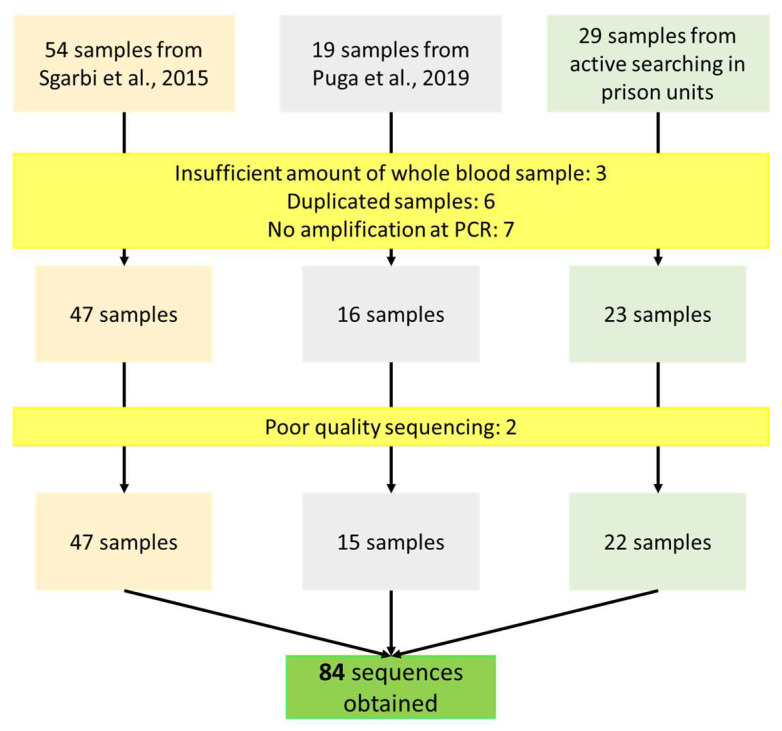
Flowchart of obtaining HIV-1 sequences from infected prisoners in this study [6,24].

Inclusion criteria were: (a) a confirmed diagnosis for HIV-1; (b) being a prisoner over 18 years old; (c) having signed the informed consent form in earlier surveys, which predicted storage of samples and their utilization in future research; (d) having sample stored in sufficient quantity to perform the analyses proposed and (e) quality of samples for sequencing. Duplicated samples and those that resulted in poor quality of sequencing were excluded, as depicted in the flowchart (Figure 1).

This study was approved by the Ethical Committee on Human Research of the Federal University of Mato Grosso do Sul in 2018 (number 2579920, CAAE 82502717.6.0000.0021), which is in accordance with the Declaration of Helsinki.

### 2.2. Amplification of HIV-1 PR/RT Region

Proviral DNA was extracted from 200 µL of each whole blood sample by using the QIAamp DNA Blood Mini kit (Qiagen, Hilden, Germany) according to the manufacturer’s protocol. The partial *polymerase* (*pol*) gene including the protease/reverse transcriptase (PR/RT) region was amplified by nested polymerase chain reaction (PCR) using the combinations of primers previously described [25].

The amplified products were analyzed by electrophoresis using agarose gels (1%). Amplicons were purified using the Exosap IT (Thermo Fischer Scientific, MA, USA), following the manufacturer’s recommendations. The purified DNA was sequenced using Big Dye Terminator Cycle Sequencing Ready Reaction kit v.3.1 (Applied Biosystems, Waltham, Massachusetts, USA) and processed with an automated ABI 3130xl sequencer (Applied Biosystems), by Sanger’s method.

### 2.3. Sequence Analysis

The sequences were edited in DNASTAR software and then aligned with reference sequences from Los Alamos HIV Sequence Database (https://www.hiv.lanl.gov (accessed on 25 March 2021)) using the Clustal W program implemented in MEGA 7.0 software, Philadelphia, PA, USA [26]. All sequences were submitted to GenBank under access numbers ON423212–ON423295. The final PR/RT alignment covered a fragment of 1261 bp, corresponding to nucleotides 2254 to 3514 position relative to the HXB2 genome.

Sequences were submitted to the REGA Subtyping Tool v3.0 program [27] and COMET [28]. Due to possibly recombinants or sequences with conflicting results, the classification was confirmed by molecular phylogeny by the Neighbor-Joining method [29] using MEGA 7.0 software [23]. Subtype reference sequences were obtained from Los Alamos HIV Sequence Database (http://www.hiv.lanl.gov/ (accessed on 25 March 2021)). Subtype assignment was based on high support clustering with reference sequences, in the bootstrap test (1000 replicates) [30]. Recombinant profiles were inferred by bootscan analyses with a sliding window of 300 bp, steps of 10 bp, and the Kimura-2 parameters model using SimPlot 3.5.1 software [31].

Those sequences that clustered together with high bootstrap support (>90) in the NJ tree were analyzed for the occurrence of possible transmission clusters. Therefore, such sequences were submitted for analysis using the nucleotide Basic Local Alignment Search Tool (BLASTn) [32] to recover reference sequences with high similarity (>95%). These sequences retrieved were added to two new alignments from pure subtypes (B and C) that were also incremented by the inclusion of Brazilian reference sequences. Afterward, the Maximum Likelihood (ML) phylogenetic tree was reconstructed with the PhyML 3.0 program using an online web server [33], to verify the maintenance of the transmission clusters according to their subtypes. The phylogenetic trees were visualized using FigTree v. 1.4.4 (http://tree.bio.ed.ac.uk/software/figtree/ (accessed on 1 June 2021)).

For subtype B, at least ten representative sequences from each Brazilian State and all sequences from MS available at the Los Alamos HIV Sequence Database were included. For subtype C analyses we included all available Brazilian reference sequences (on 14 July 2021). Before performing the phylogenetic analyses to confirm the transmission clusters, drug-resistance mutation positions were stripped from each alignment, resulting in a fragment of 900 bp from nucleotides 2262 to 3260 relative to the HXB2 genome. The Smart Model Selection recommended the GTR+I+G nucleotide substitution model to be used in the ML [34]. The heuristic tree search was performed using the SPR branch-swapping algorithm, and the branch support was calculated with the approximate likelihood-ratio (aLRT) SH-like test [35]. Transmission cluster classification was defined based on aLRT (>90) in the phylogenetic analyses, and low mean pairwise genetic distances (≤4.5) of clustered sequences have been employed. In this study, pairs (n = 2) and larger clusters (n ≥ 3) are all referred to as clusters.

### 2.4. Genotypic Analysis of HIV-1 Drug Resistance

To investigate the presence of transmitted drug resistance mutations (TDRM), the sequences were submitted to Stanford HIV Database for Transmitted DRM [TDRM/Calibrated Population Resistance Tool (CPR Tool)] Version 6.0 [36], which uses the mutation list according to Bennett et al., 2009 [37]. For treatment-experienced individuals, acquired drug resistance mutations (ADRM) were assessed by Stanford HIV Drug Resistance Database [38].

### 2.5. Statistical Analysis

Statistical analysis was conducted using the STATA 13.0 software (Stata Corporation, College Station, TX, USA). Median, standard deviation (SD), range, and frequencies (%) were used to describe patients’ characteristics. The frequency of DRMs was also calculated, and the chi-square test or Fisher’s exact test was employed when appropriate. Variables with a *p*-value of 0.20 or less were included in the logistic regression backward stepwise model. A *p*-value of < 0.05 was defined as statistically significant. Odds ratios and 95% confidence intervals (CI) were used as the measure of the strength of the association between outcome and independent variables.

## 3. Results

The mean age of the 84 studied subjects was 35.8 years (SD 9.27), ranging from 19 to 56 years. As the prison population is composed mainly of men, 76 individuals were male (90.5%) and only 8 (9.5%) were female. Concerning individual original residence, 64% of them were from the Midwest region, however, 59.5% of the individuals were born in another Brazilian state, but were detained in prisons in MS. More than half of the participants were non-white (63.0%), heterosexual (83.3%), and reported a low level of formal education (equal or less than 9 years of schooling—92.8%).

**Figure 2:**
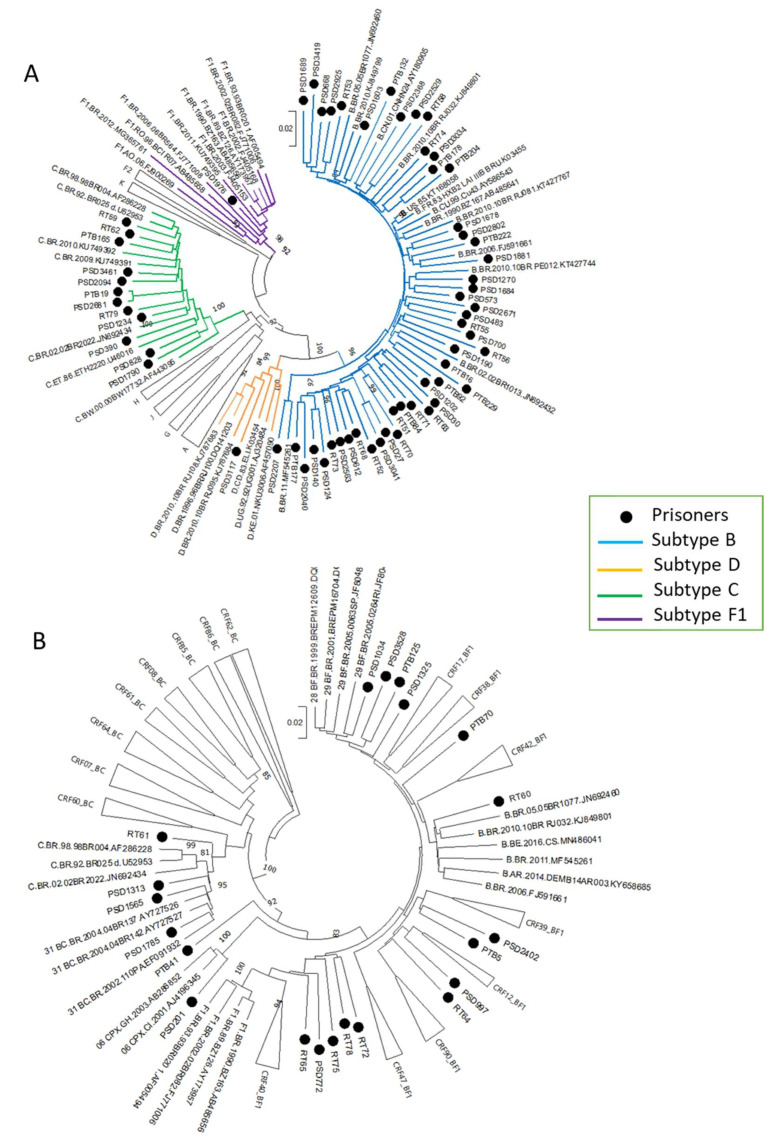
Neighbor-Joining (NJ) phylogenetic tree of 84 HIV-1 PR/RT sequences from prisoners from Mato Grosso do Sul, Central-West Brazil. The analyzed PR/RT alignment covered a fragment of 1261 bp, corresponding to nucleotides 2254 to 3514 relative to HXB2 genome. Sequences from this study are marked with a full circle. Bootstrap values are represented only if >80. (**A**) Pure HIV-1 subtypes, involving 120 HIV-1 PR/RT sequences and (**B**) Recombinant sequences, involving 96 HIV-1 PR/RT sequences.

A total of 102 subjects were recruited, proviral DNA was extracted from whole blood samples of 100 individuals for whom we had biological material stored, and partial polymerase fragment was amplified by nested-PCR in 93 of them. Seven samples were excluded for being double sampled and two due to poor sequencing quality. Therefore, molecular results from 84 sequences were available for the following analysis (Figure 1). Phylogenetic analyses revealed that the HIV-1 subtype B was the most prevalent clade in our sampling, as 49 (58.3%; 95% CI: 47.3–68.6) were classified as subtype B, followed by C (n = 12; 14.3%), and two sequences classified as subtype D and F1 each one (Figure 2A). Twenty-one sequences (25.0%; 95% CI: 16.8–35.6%) were possible recombinants and were checked by phylogenetic and bootscan analyses. These analyses revealed that four presented circulating recombinant forms (CRFs) like patterns as follows: two CRF31_BC (2.4%, PSD1565, and PSD1785), one CRF12_BF (1.3%, PSD997), and one CRF28/29_BF (1.3%, PSD1034). Seventeen (20.2%) were unique recombinant forms (URFs), including one URF 06_CPXG (1.3%, PSD201), three URF_BC (3.6%, PSD1313, PTB41, and RT61), one URF_BD (1.3%, RT60), and twelve URF_BF (14.3%, PSD772, PSD1325, PSD2402, PSD3528, PTB5, PTB70, PTB125, RT64, RT65, RT72, RT75, and RT78) (Figure 2B).

Considering only the phylogenetic analysis, a total of 33.3% (28/84) of the sequences were found to be linked to at least one another sequence grouped into 17 distinct clusters (Table 1). After the combination of phylogenetic and distance analysis, the possible transmission clusters (2A, 2B, 5, 6, 9, 10, and 11) were not confirmed. Some of the originally detected clusters remained with the same configuration; meanwhile, some of them presented a new shape. The remaining 14 clusters were detected including sequences from other prisoners or sequences obtained from the Los Alamos sequence database, ranging from 2 to 6 sequences per cluster, with a mean of 3.5 sequences per cluster and an overall mean genetic distance in each cluster ranged from 0.027 to 0.044. Twelve clusters involved HIV-1B sequences and one, HIV-1C. Sociodemographic and behavioral characteristics according to clustering are listed in Table 2. Having a history of homosexual contact increases the chance of forming a cluster by 9.38 times (95% CI 1.59–55.25, *p* = 0.01), and being infected by a non-HIV-1B subtype is a protective factor for clustering (Table 2), however, this result should be interpreted with caution due to the high CI 95% observed.

Among HIV-1 subtype B samples, 17 of 49 sequences isolated (32.7%) were involved in clusters. These clusters were formed by prisoners’ sequences and other studies from MS available in Los Alamos Sequence Database and obtained previously by these studies conducted among different population groups such as patients from reference centers for HIV-1 treatment, men who have sex with men (MSM), and prisoners [36,37,38]. Most HIV-1B clusters (7/13; 3.8%) contained more than two sequences. The two largest clusters were formed by 6 samples each (clusters 1 and 4C), involving prisoners, heterosexual men, MSM, and women from the community. The other three clusters were formed by five sequences each, two of them composed of males and a female (clusters 3 and 4A), and one composed only by men (cluster 14). In cluster 14, four out of five men had a history of imprisonment, and one was MSM. The two clusters containing three sequences each were formed by male prisoners and MSM from the community (clusters 4B and 7).

Six clusters (6/14; 42.9%) contained two sequences each. Three of them contained a male prisoner and a female from the community (clusters 2C, 12, and 13), from the remaining three, one was male-only and two of them were formed by a prisoner and a male from the community (clusters 8 and 16) and another one, was formed by two prisoners from the same penal institution (cluster 15). Interestingly, no sequences from the other Brazilian States were clustered together with MS’ sequences of HIV-1B.

Only one cluster composed by two study samples (2/12; 16.7%) and two others sequences obtained from Los Alamos sequence database was formed in the HIV-1 subtype C analysis. Of these, one was an MSM from MS and the other is a sequence collected in 2006 in Rio de Janeiro state, whose epidemiological data is lacking.

Regarding antiretroviral treatment, most (50/84; 59.5%) of individuals were treatment-experienced, while 40.5% (34/84) were ART-naïve. Resistance analysis of PR and RT sequences from the 50 ART-experienced samples showed an alarming prevalence of ADRM, since 16/50 (32.0%) of them had at least one DRM. The distribution of resistance mutations by drug-class reflected the predominance of non-nucleoside reverse transcriptase inhibitors (NNRTIs:14/50; 28%), followed by nucleoside reverse transcriptase inhibitors (NRTI: 6/50; 12%), and protease inhibitors (PI: 1/50; 2%). Of these, eleven were singleton mutations, and five, multiple. The most DRM detected was K103N to NNRTI (9/15). Meanwhile, TDRM were detected in 2/34 (5.9%) ART-naïve samples, one of them presented only K103N, and another accumulated three mutations, K219Q, K103N, and L90M (Table 3).

## 4. Discussion

Particularly in developing countries, prisons have been known as hotspots of several pathogens, including HIV-1, as a result of overcrowding, insufficient health assistance, and prisoners’ high-risk behaviors [5,39]. Considering that there are a lack of studies regarding this theme in the prison population, in this study we analyzed HIV-1 pol sequences isolated from 84 prisoners’ blood samples collected between 2013 and 2018 in twelve prisons in MS, Brazil, exploring molecular and epidemiological data.

To our knowledge, there is a single study evaluating HIV infection features in the prison population from Central-West Brazil [39]. It was conducted more than ten years ago in prisons from Goiás and MS States and had a sample size of 27 prisoners, three times smaller than ours. Additionally, herein, we evaluate further elements, like molecular transmission clusters, DRM, and HIV-1 subtyping, comparing prisoner’s sequences and others Brazilian sequences available in Los Alamos Sequence Database.

Considering the data published so far, relevant data on the molecular epidemiology of HIV-1 in Brazilian prisons are scarce, thus limiting comparisons. In our sampling, HIV-1 subtype B was the most prevalent (58.3%; CI 95%: 47.3–68.6). This finding is consistent with studies in the general population from the same region, ranging from 65.3 to 69.9% [40,41,42], and in prisoners from the Central-West region (48%) [39]. Therefore, the proportion of recombinant forms in this study was higher (25%) than the one found in the general population from the same State (13.3%), suggesting HIV-1 genetic complexity among prisoners (*p* = 0.002) [41].

For HIV-1, genetic diversity accumulates rapidly, making it conceivable to infer the viral transmission network using phylogenetic reconstruction. Hence, it is possible to describe feasibly the history of infections of these closely related viruses in a phylogenetic tree, assuming that the hosts are connected by a common source, a direct or a short chain of transmissions, especially when detailed epidemiological data is available [23]. In this study, we analyzed the structures of the transmission clusters through the integration of molecular and sociodemographic data. We found that 22.6% of the prisoners were grouped into 15 molecularly defined HIV transmission clusters, indicating that most HIV-1 subtype B infections in prisons were linked to a cluster that might be associated with local HIV-1 networks in and outside the prison. These results reinforce the hypothesis that the HIV molecular epidemics inside prisons are similar to those observed in MS’ general population, a result of the intense flow between the prison and the community.

One of the clusters (cluster 15) involved two individuals imprisoned in the same prison facility, suggesting an epidemiological link between them. In addition, we described clusters involving individuals with different HIV risk behaviors, such as MSM and prisoners (clusters 3, 4A, 4B, 4C, 7, 16, and 17), and others with no commonly identifiable type of exposure. This supports the idea that the HIV-1 variants circulating in the prison environment may influence the maintenance of their circulation and dissemination in the community. Thus, preventive measures, better opportunities for diagnosis, and adequate treatment of prisoners with HIV can have effects not only on the quality of life and survival of these individuals but also on the community.

The present study for the first time investigates the transmission networks in prisoners from MS State and verified the increased possibility to be involved in a cluster when belonging to subtype C and having homosexual contact. Since subtype C prevalence in MS is 14.3% in the present study and 8% in our previous study [41], the formation of networks involving this subtype will be more easily verified than among subtype B which has a high prevalence. Some factors related to the prison environment that could be directly associated with high HIV prevalence in prisoners, such as the high number of individuals in the same cell, previous incarceration, and more prolonged incarceration; however, based on the present data they were unrelated to cluster formation.

The high prevalence of individuals who were not on antiretroviral treatment at the time of blood and data collection (40.5% ART-naïve) also demonstrates that opportunities for diagnosis and treatment are being missed, a reflection of the failure of health policies in Brazilian prisons. Furthermore, a previous study in the same prison facilities showed that, for inmates with a confirmed diagnosis of HIV-1 infection for whom free treatment is offered, approximately half of them presented viral load > 200 copies/mL [5], evidencing low adherence to treatment or indicating virological failure, which may be suggestive of the existence of DRM.

Regarding ADRM, 32.0% of treatment-experienced prisoners had at least one DRM, thus pointing out poor treatment adherence, which is common among high-risk behavior groups. TRDM were found in 5.9% of the prisoners, in a setting where pre-treatment genotyping is not a routine, as the Brazilian Ministry of Health recommends it only in specific cases. A study conducted in southern Brazil showed 24% of transmitted and acquired resistance mutations to antiretroviral drugs and an alarming proportion of virologic failure in prisoners on antiretroviral treatment [7].

The mutation K103N was found in 11 prisoners (13.1%), 9 as an ADRM and 2 as a TDRM. This substitution may cause high-level reductions in nevirapine and efavirenz susceptibility. These drugs are not part of first-line antiretroviral treatment in Brazil for all person living with HIV/aids. However, it becomes a concern since efavirenz is part of the preferred regimen (tenofovir, lamivudine, and efavirenz) in the treatment of individuals coinfected with tuberculosis (TB) [8]. TB is highly prevalent in Brazilian prisons, mainly due to the prison environment driving TB incidence [43]. The presence of K103N prior to therapy in treatment-naive individuals is correlated with an increased risk of virologic failure of these efavirenz-containing triple-drug regimens [44].

This study has some limitations. We interviewed all individuals face-to-face; consequently, risk behaviors may have been under-reported, leading to potential underestimation of associations with these variables and DRM prevalence. This research comprises not only individuals with epidemiological features in common but also the spread of strains between different key populations, like MSM and prisoners. We highlight that HIV-1 may be circulating not only behind the bars but also in the community outside them. So, it can help to understand that public health interventions in different scenarios, like prisons, may affect the whole epidemic.

We acknowledge that our sample size and sampling are not representative of the entire prison population in Brazil. However, the remarkable HIV-1 genetic diversity, the diverse recombination patterns, the high rate of acquired resistance, and the possible transmission links among prisoners and individuals from the general population described in this study raise important public health concerns. Moreover, our data highlight the need for routine surveillance of HIV-1 infection including resistance genotypic tests for prisoners by public health institutions. Considering the high disease burden and risk behaviors inside prisons and the dynamic relationship of prisoners with the outside community, great benefits could be achieved from specific programs aimed at STI, especially HIV prevention, counseling screening, and treatment.

## Figures and Tables

**Table 1:** Transmission clusters among HIV-1 prisoners’ sequences according to aLTR and genetic distance.

Cluster	aLRT	Sequences	Distance	Subcluster	aLRT	Distance	Cluster Confirmation
HIV-1B							
1	0.99	BRMS02.JF342274	0.034	-	-	-	Confirmed
		BRMS46.JF342297					
		HET801.MF545297					
		HET462.MF545270					
		HET631.MF545285					
		RT74					
2	0.94	PSD1689	0.054	2A	0.939	0.053	Not confirmed
		BRMS53.JF342303					
		BREPM1028.EF637053					
		07CE140556.KY581465					
		PSD668		2B	0.71	0.049	Not confirmed
		HET830.MF545301					
		PSD3419		2C	0.968	0.039	Confirmed
		HET640.MF545286					
3	0.92	HSH874.MF545336	0.036				Confirmed
		HET814.MF545299					
		RT53					
		HSH881.MF545340					
		HET249.MF545255					
4	0.90	HET622.MF545284	0.052				
		HET850.MF545302					
		PTB204		4A	0.91	0.027	Confirmed
		BRMS99.HQ831455					
		BRMS97.HQ831454					
		HSH871.MF545333					
		PTB178					
		PSD3034		4B	0.96	0.043	Confirmed
		HSH408.MF545314					
		HSH862.MF545331					
		HET738.MF545293					
		HET521.MF545276					
		HET323.MF545260					
		HSH719.MF545326					
		HET437.MF545267					
		HSH717.MF545325		4C	1	0.039	Confirmed
		MF545324.HSH716					
		PSD2529					
		HSH875.MF545337					
		MF545332.HSH867					
		HET757.MF545294					
5	0.91	BREPM1084.FJ195088	0.051	5A	0.91	0.051	Not confirmed
		PTB16					
		HET219.MF545251		5B	0.784	0.052	Not confirmed
		PSD1190					
6	0.91	RT58	0.074	-	-	-	Not confirmed
		02BR002.DQ358805					
7	0.97	PSD1678	0.044	-	-	-	Confirmed
		BRMS117.HQ831457					
		HSH859.MF545329					
8	1	PSD2207	0.044	-	-	-	Confirmed
		HET362.MF545261					
9	0.93	PTB92	0.057	9A	0.095	0.057	Not confirmed
		HET446.MF545269					
		PTB229					
		HET522.MF545277					
		PSD700		9B	0.73	0.044	Not confirmed
		HET479.MF545271					
10	0.98	RT56	0.072	-	-	-	Not confirmed
		BRTO12 83.GU214093					
11	0.912	RT51	0.054	-	-	-	Not confirmed
		RT70					
12	1	RT52	0.037	-	-	-	Confirmed
		BRMS27.JF342282					
13	0.92	RT68	0.037	-	-	-	Confirmed
		HET501.MF545272					
14	0.91	RT73	0.036	-	-	-	Confirmed
		PSD3041					
		HET671.MF545288					
		PSD27					
		HET438.MF545268					
15	0.97	PSD124	0.042	-	-	-	Confirmed
		PSD140					
16	0.93	RT71	0.031	-	-	-	Confirmed
		HSH239.MF545312					
HIV-1C							
17	0.964	PTB19	0.021	-	-	-	Confirmed
		HSH880.MF545237					
		PSD2681					
		06BRRJ_09.KF255844					

**Table 2:** Sociodemographic and behavioral characteristics of HIV-infected prison inmates according to clustering (n = 84), Central Brazil.

Variable	N	(%)	Clustered N (%)	Not Clustered N (%)	OR (95% CI)	*p*	aOR (95%CI) ^ˠ^	*p*
Total			19 (28.4)	48 (71.6)	(18.7–40.6)			
Gender								
Male	60	(89.5)	19 (31.7)	41 (68.3)	-	-	-	-
Female	7	(10.5)	0 (0.0)	8 (100.0)	-	-	-	-
Age (years)								
18–34	31	(46.3)	11 (35.5)	200 (64.5)	1.0			
35 or more	36	(53.7)	8 (22.2)	28 (77.8)	0.52 (0.17–1.52)	0.23	-	-
Skin colour/ethnicity								
White	22	(33.3)	10 (45.5)	12 (54.5)	1.0		1.0	
Non-white	44	(66.7)	9 (20.4)	35 (79.6)	0.31 (0.10–0.94)	0.04	0.34 (0.09–1.25)	0.10
Educational (years) ^#^								
0	30	(46.8)	7 (23.3)	23 (76.7)	-	-	-	-
1–12	31	(48.4)	11 (35.5)	20 (64.5)	-	-	-	-
>12	3	(4.7)	0 (0.0)	3 (100.0)	-	-	-	-
Naturality								
MS State	41	(61.2)	13 (31.7)	28 (68.3)	1.0			
Other	26	(38.8)	6 (23.1)	20 (76.9)	0.71 (0.24–21.2)	0.4	-	-
Persons in the same cell								
1–10	27	(40.9)	8 (29.6)	19 (70.4)	1.0			
11–30	22	(33.3)	6 (27.3)	16 (72.7)	0.89 (0.22–2.52)	0.86	-	-
31 or more	17	(25.7)	5 (29.4)	12 (70.6)	0.98 (0.08–1.60)	0.98	-	-
Time of incarceration ^#^								
Up to 5 years	50	(76.9)	14 (28.0)	36 (72.0)	1.0			
More than 5 years	15	(23.1)	5 (33.3)	10 (66.7)	0.69 (0.37–4.43)	0.69	-	-
Previous incarceration								
No	19	(28.4)	5 (26.3)	14 (73.7)	1.0			
Yes	48	(71.6)	14 (29.2)	34 (70.8)	1.15 (0.35–3.81)	0.81	-	-
Illicit drug use								
No	1	(1.5)	0 (0.0)	1 (100.0)	-	-	-	-
Yes, no injecting drugs	63	(97.0)	9 (14.3)	54 (85.7)	-	-	-	-
Yes, injecting drugs	1	(1.5)	0 (0.0)	1 (100.0)	-	-	-	-
Cocaine base pasta use ^#^								
No	55	(84.6)	13 (23.6)	42 (76.4)	1.0			
Yes	10	(15.4)	6 (60.0)	4 (40.0)	4.84 (1.18–19.8)	0.02	3.57 (0.69–18.4)	0.12
History of homosexual contact ^#^								
No	51	(19.1)	11 (21.6)	40 (78.4)	1.0			
Yes	12	(80.9)	7 (58.3)	5 (41.7)	4.90 (1.43–16.82)	0.01	8.03 (1.30–49.23)	0.02
Number of sexual partners in the last 12 months								
0	15	(22.4)	4 (26.7)	11 (73.3)	1.0			
1-5	43	(64.2)	11 (25.6)	32 (74.4)	0.94 (0.24–3.59)	0.93	-	-
≥6	9	(13.4)	4 (44.4)	5 (55.6)	2.20 (0.38–12.6)	0.37	-	-
Use of condoms in the last 12 months								
Always	29	(43.9)	9 (31.0)	20 (69.0)	1.0			
Occasionally/Never	37	(56.1)	10 (27.0)	27 (73.0)	0.82 (0.29–2.39)	0.72	-	-
History of STI								
No	30	(45.5)	10 (33.3)	20 (66.7)	1.0			
Yes	36	(54.5)	9 (25.0)	27 (75.0)	0.7 (0.23–1.95)	0.46	-	-
Genital ulcers								
No	62	(92.5)	16 (25.8)	46 (74.2)	1.0			
Yes	5	(7.5)	3 (60.0)	2 (40.0)	4.31 (0.66–28.2)	0.12	-	-
HIV-1 Subtype								
B	49	(73.2)	17 (34.7)	32 (65.3)	1.0			
Non-B	18	(26.8)	2 (11.1)	33 (88.9)	0.23 (0.04–1.14)	0.05	0.01 (0.01–1.7)	0.05

OR: odds ratio; CI: confidence interval; aOR: adjusted OR; STI: sexually transmitted infections; STI: Sexually transmitted infection; MS: Mato Grosso do Sul, Central Brazil. Statistically significant (*p* < 0.05). ^#^ The total N corresponds to those who answered the questions. ˠ Adjusted for variables with a *p*-value of 0.20 or less on univariate analysis.

**Table 3:** Characteristics of the 18 prisoners with DRM, Central Brazil, 2013–2018.

ID	Age	Resistance Mutations			HIV-1 Subtype	Type of Exposure
		NRTI	NNRTI	PI		
ART-experienced n = 16						
PSD1270 *	28	M184I			B	Sex with HIV-infected partner
PSD2402	30		K103N		URF_BF	Homemade tattooing, multiple sexual partners
PSD2529	27		K103NS, V106M		B	Irregular condom use, sharing of sharp objects
PSD2671	41		K103N		B	Sex with HIV-infected and drug user
PSD2802	37		V106I		B	Multiple sexual partners
PSD3034	40	M41L, M184V, T215Y	K103N, P225H		B	Sex with drug user
PTB16	27		K103N		B	None
PTB70	26		V106I		URF_BF	MSM, Irregular condom use, sharing of sharp objects
PTB165	26	E44D			C	MSM, Irregular condom use, Sex with drug user, sharing of sharp objects
PTB177	54		Y181YH		B	Irregular condom use, sharing of sharp objects
PTB204	35	M41L, L74I, M184V, T215Y	K103N, E138A, P255H		B	MSM and sex with drug user
PTB229	44	A62V, K65R, M184V	L100I, K103N		B	None
RT58	27	D67NK70R, T215Y	K103N, V106I, V179T, Y188F, H221Y	I84V, L90M, Q58E, G73S	B	Irregular condom use, MSM and sex with drug user
RT64	28		Y181F		URF_BF	Irregular condom use
RT71	44		F227FL		B	MSM, sharing of sharp objects
RT75	27		K103N		URF_BF	MSM, history of STI
ART-naive n = 2						
PSD2368	26		K103N		B	Sharing of sharp objects
PSD3117	47	K219Q	K103N	L90M	D	History of STI

ID: sample identification; NRTI—Nucleoside Reverse Transcriptase Inhibitor, NNRTI—Non-Nucleoside Reverse Transcriptase Inhibitor; PI—Protease inhibitor; MSM: men who have sex with men; STI: Sexually transmitted infection. URF—unique recombinant form. * Female prisoner.

## Data Availability

All relevant data are within the manuscript.
